# Supplementary material for: Using geographic information systems to link population estimates to wastewater surveillance data in New York State, USA
Source: PLOS Glob Public Health. 2023 Jan 26;3(1):e0001062. doi: 10.1371/journal.pgph.0001062 (PMC10021809; doi:10.1371/journal.pgph.0001062)
Supplement: S1 Appendix — (DOCX) [file pgph.0001062.s001.docx]

S1 Appendix: Details on sewershed boundary creation

# Additional notes on methods

## Digitized from address list

NY State Tax Parcel database was used to estimate parcels served by this sewer system. First, parcels within the county of the wastewater treatment plant (WWTP) were selected if they were on public sewer. Then, these parcels were subset into parcels on the streets identified in the address list. Last, the range of house numbers and address numbers were selected that matched the address list provided. The final set of parcels were assigned to the sewershed to draw the boundary.

## Digitized from manhole/sewer main shapefile

Treatment plant operators were contacted with a request for data. The operator provided a digital shapefile that contained locations of manholes and/or sewer mains linked to the WWTP. These data were then overlaid on to the tax parcel data for public sewers within the county. Parcels that intersected with sewer mains, overlapped with manholes, and were adjacent to these data were selected to draw the final boundary for the WWTP sewershed.

## Digitized from physical map (JPEG/PDF)

Treatment plant operators that did not have digital data provided copies of physical maps to ur research team including photos of maps and PDF scans or copies of maps showing: sewer service areas, sewer districts, and/or manhole and sewer main locations. NY State Tax Parcel data for the county that the WWTP was within were then subset for parcels on public sewer. Then, additional data including town and village boundaries and primary roads from the US Census database were added to orient the data and allow for cross-reference with the map. Then, parcels that matched the boundary provided in the map were selected to form the final polygon for the WWTP.

## Parcel digitized from DANC records

Development Authority of the North County (DANC) provided a shapefile of sewersheds that were created starting in 2010 and updated annually. The version this project used was obtained in 2021. Boundary estimates provided were overlaid with NY State Tax Parcel data for public sewers in the counties provided by DANC (Jefferson, Lewis, and St. Lawrence Counties). These parcles were compared to the boundaries provided by DANC. If the boundary matched, then the DANC boundary was used to create the WWTP sewershed polygon. If the boundary was missing some parcels adjacent to the DANC polygon, then those parcels were combined with the DANC polygon to create the final WWTP sewershed.

## Parcel digitized from description

Treatment plant operator provided a text description of the service area including roads, natural boundaries, number of influent points, and towns/villages/cities served. The boundary was drawn using ArcGIS software and Census data for major roads, municipal boundaries, and NY State Tax Parcel database. Tax parcels that were recorded as paying a sewer tax and fall within the described service area were assigned to the sewershed.

## Parcel digitized no description

New York State Tax Parcel database was used to estimate the parcels served by this sewer system. The coordinates of the WWTP were mapped using Arc GIS and then public sewer parcels for the county that the WWTP was within were added. Parcels that were clustered around the point were then assigned to that WWTP to create the sewershed polygon. If a WWTP was adjacent to the edge of a county, then both county’s parcel data were used. In some cases, this meant that the boundary for the treatment plant crossed county lines. Sometimes WWTPs adjacent to county borders only had public sewer parcels that were within the same county and did not cross the boundary.

There were also cases were WWTPs were close to one another and public sewer parcels were adjacent to each other with no obvious clustering to distinguish where one sewershed ended and another began. In these instances, an additional dataset was used for the parcel data know as the Special Districts table. These tables provided information on each parcel such as fire districts, water districts, and sewer districts. These data were joined to the spatial data by parcel ID and selected for sewer districts. In some cases, this provided clear distinction between sewer districts and the WWTP they were linked to. In other cases, there were more sewer districts than WWTPs. Therefore, the municipality was contacted to learn what districts corresponded to the correct treatment plant. Then, those districts were combined to form the final polygon boundary.

## Provided by treatment plant/municipal/county

Some treatment plants were able to provide exact boundary data to our team in the form of digital shapefiles. In these instances, the data provided were used to form the boundary of the WWTP’s sewershed and directly added to the database without modification.

## Village boundary

For sites with no clear clusters of parcels that were on public sewer and contact with the treatment plant or village government could not be established, the municipal boundary for the village from the US Census was used.

# List of websites/resources by county and permit (if applicable)

Some of the data used to create the sewershed polygons in the final database came from publicly available websites such as municipal or county websites. In this section, we provide lists of data sources used in this project. If a county is not in this list, no data from public websites were found for this project and other data sources listed in the main document were used.

## Albany

Sanitary Sewer District Boundary. Town of Bethlehem – County of Albany – State of New York. (2014). Accessed July 12, 2022.

<https://www.townofbethlehem.org/DocumentCenter/View/4286/Sewer-District-Boundary-Map---2014?bidId=>

Town of Bethlehem Sanitary Sewer Location Map. (2009). Accessed July 12, 2022.

<https://www.townofbethlehem.org/DocumentCenter/View/3757/Appendix-C---Location-of-Water-and-Sewer-Service-Maps?bidId=>

Town of Bethlehem document center. (2022) *Town of Bethlehem, NY.* Accessed July 12, 2022. <https://www.townofbethlehem.org/DocumentCenter/>

## Allegany

Built Environment: Existing and Planned Sewer Service Areas. (n.d.) Accessed July 13, 2022. *Allegany County, New York: GIS/County Mapping.* <https://www.alleganyco.gov/departments/planning/giscounty-mapping/>

## Broome

Broome County Sewered Areas. (2003). Broome County Department of Planning and Economic Development. Accessed December 11, 2021. <https://gis.broomecountyny.gov/Website/GISWeb/maps/seweredareas.pdf>

## Cattaraugus

Final NPDES Permit for Salamanca Wastewater Treatment Plant in New York. (2019). Accessed July 13, 2022. <https://www.epa.gov/npdes-permits/final-npdes-permit-salamanca-wastewater-treatment-plant-new-york>

## Dutchess

CPL (2020). Map Plan Report. Town of LaGrange Manchester Sewer District. CPL Project No. 12977.24. Accessed July 13, 2022. <http://www.lagrangeny.gov/CMSJavascripts/pwMSDmprJuly2020.pdf>

## Greene

Delaware Engineering, D.P.C. (2016). Map Book of Focus Parcels. *Green County Infrastructure Inventory and Development Project.* Accessed July 13, 2022. <https://www.greenegovernment.com/wp-content/uploads/2019/05/Greene-Co.-Municipal-Maps-with-Focus-Parcels-2016.pdf>

## Nassau

Long Island Index Map. (2022). *Long Island Index*. Accessed July 13, 2022. <http://www.longislandindexmaps.org/>

## New York city

Open Sewer Atlas NYC. (n.d.) Accessed July 13, 2022. <https://openseweratlas.tumblr.com/>

Open Sewer Atlas NYC. (2022). NYC Open Data. Accessed July 13, 2022. <https://opendata.cityofnewyork.us/projects/open-sewer-atlas-nyc/>

Sewer features and drainage areas in NYC. (2016). Open Sewer Atalst NYC: Arc GIS. Accessed July 13, 2022. <https://www.arcgis.com/home/item.html?id=be88972b0e374879adc6173e67236365>

## Oneida

East Oneida Lake Wastewater Treatment Plant. (2017). Village of Sylvan Beach. Accessed July 13, 2022. <https://www.villageofsylvanbeach.org/dept-eol>

## Rockland

Sewer Districts – Rockland County, New York. (2010). Accessed December 12, 2021. <https://www.rocklandgis.com/portal/sharing/rest/content/items/2d019f2d21264d73ac2c21fc567e3787/data>

## Suffolk

Cartography and GIS (2022). Planning and Environment: Suffolk County Government. Accessed July 13, 2022. <https://suffolkcountyny.gov/Departments/Economic-Development-and-Planning/Planning-and-Environment/Cartography-and-GIS#gis>

Planning and Environment (2022). Town of Huntington – Long Island, New York. Accessed July 13, 2022. <https://www.huntingtonny.gov/planning-environment>

Sanitary Main Line. (2021). Sufflk County Open Data. Accessed July 13, 2022. <https://opendata.suffolkcountyny.gov/search?q=sewer>

## Tompkins

Campbell, C. (2021). Sewer Areas. Tompkins County Open Data. Accessed July 13, 2022. <https://tcdata-tompkinscounty.opendata.arcgis.com/datasets/tompkinscounty::sewerareas/about>

## Westchester –

Municipal Sewer Mains. (2022). Westchester County GIS. Accessed July 13, 2022. <https://gis.westchestergov.com/datasets/0fe25fc84ae441ac85dff3d1c3828dc6_152/explore>

County Sewer Districts. (2022). Westchester County GIS. Accessed July 13, 2022. <https://gis.westchestergov.com/search?collection=Dataset&q=sewer>
