# Supplementary material for: Using geographic information systems to link population estimates to wastewater surveillance data in New York State, USA
Source: PLOS Glob Public Health. 2023 Jan 26;3(1):e0001062. doi: 10.1371/journal.pgph.0001062 (PMC10021809; doi:10.1371/journal.pgph.0001062)
Supplement: S1 Table — (DOCX) [file pgph.0001062.s003.docx]

| S1 Table: Countywide summary information for urban and rural counties | | | | | | | | | |  |
| --- | --- | --- | --- | --- | --- | --- | --- | --- | --- | --- |
| *County* | *Rural or urban* | *Number of sewers* | *Median number of people in each sewer system* | *Median sewershed population density* | *Median sewershed area (km^2^)* | *Median WWTP discharge capacity* | *Total county population* | *Total population on sewer* | *Proportion of county population on sewer* | |
| Albany | Urban | 13 | 1,809.0 | 423.371 | 4.273 | 0.420 | 304,204.0 | 277,996.0 | 0.914 | |
| Allegany | Rural | 9 | 1,162.0 | 368.481 | 2.859 | 0.290 | 48,946.0 | 16,294.0 | 0.333 | |
| Bronx | Urban | 2 | 983,124.0 | 17003.403 | 61.857 | 237.500 | 1,385,108.0 | 1,385,108.0 | 1.000 | |
| Broome | Urban | 9 | 696.0 | 294.951 | 3.240 | 0.110 | 200,600.0 | 122,015.0 | 0.608 | |
| Cattaraugus | Rural | 12 | 711.0 | 303.386 | 3.228 | 0.180 | 80,317.0 | 30,198.0 | 0.376 | |
| Cayuga | Rural | 7 | 1,441.0 | 426.808 | 3.141 | 0.330 | 80,026.0 | 39,511.0 | 0.494 | |
| Chautauqua | Urban | 15 | 1,541.0 | 291.300 | 4.797 | 0.660 | 134,905.0 | 80,091.0 | 0.594 | |
| Chemung | Urban | 2 | 28,061.0 | 862.299 | 34.427 | 12.100 | 88,830.0 | 56,122.0 | 0.632 | |
| Chenango | Rural | 6 | 1,268.0 | 390.787 | 3.911 | 0.375 | 50,477.0 | 12,164.0 | 0.241 | |
| Clinton | Rural | 11 | 1,100.0 | 213.966 | 4.493 | 0.400 | 82,128.0 | 82,128.0 | 1.000 | |
| Columbia | Rural | 9 | 1,142.0 | 419.679 | 2.586 | 0.250 | 63,096.0 | 14,810.0 | 0.235 | |
| Cortland | Urban | 4 | 496.0 | 189.539 | 2.723 | 0.140 | 49,336.0 | 28,001.0 | 0.568 | |
| Delaware | Rural | 13 | 42.0 | 17.863 | 2.925 | 0.400 | 47,980.0 | 6,977.0 | 0.145 | |
| Dutchess | Urban | 24 | 1,077.0 | 459.061 | 2.340 | 0.190 | 297,488.0 | 117,007.0 | 0.393 | |
| Erie | Urban | 22 | 2,638.0 | 365.025 | 7.147 | 0.735 | 919,040.0 | 828,628.0 | 0.902 | |
| Essex | Rural | 12 | 427.0 | 121.301 | 3.419 | 0.130 | 39,370.0 | 17,197.0 | 0.437 | |
| Franklin | Rural | 7 | 690.0 | 180.071 | 4.643 | 0.485 | 51,599.0 | 16,955.0 | 0.329 | |
| Fulton | Rural | 4 | 1,104.0 | 369.368 | 3.023 | 0.135 | 55,531.0 | 25,630.0 | 0.462 | |
| Genesee | Rural | 11 | 719.0 | 454.331 | 2.676 | 0.140 | 60,079.0 | 31,029.0 | 0.516 | |
| Greene | Rural | 11 | 412.0 | 212.869 | 4.693 | 0.330 | 49,221.0 | 11,944.0 | 0.243 | |
| Hamilton | Rural | 3 | 113.0 | 38.493 | 2.339 | 0.140 | 4,836.0 | 258.0 | 0.053 | |
| Herkimer | Rural | 5 | 4,602.0 | 669.273 | 8.699 | 2.000 | 64,519.0 | 28,928.0 | 0.448 | |
| Jefferson | Urban | 22 | 682.0 | 228.842 | 2.883 | 0.130 | 116,229.0 | 46,697.0 | 0.402 | |
| Kings | Urban | 5 | 710,768.0 | 15799.424 | 52.223 | 110.000 | 2,504,700.0 | 2,504,700.0 | 1.000 | |
| Lewis | Rural | 9 | 425.0 | 213.167 | 1.243 | 0.060 | 27,087.0 | 7,259.0 | 0.268 | |
| Livingston | Rural | 10 | 2,450.0 | 401.131 | 7.398 | 0.580 | 65,393.0 | 37,019.0 | 0.566 | |
| Madison | Rural | 6 | 4,080.0 | 482.685 | 7.563 | 1.125 | 73,442.0 | 26,816.0 | 0.365 | |
| Monroe | Urban | 4 | 99,085.0 | 453.761 | 340.185 | 14.750 | 744,344.0 | 688,264.0 | 0.925 | |
| Montgomery | Urban | 5 | 2,270.0 | 458.865 | 6.122 | 2.000 | 50,219.0 | 27,584.0 | 0.549 | |
| Nassau | Urban | 9 | 24,041.0 | 1672.649 | 11.347 | 5.300 | 1,339,532.0 | 1,257,832.0 | 0.939 | |
| New York | Urban | 1 | 655,794.0 | 28190.580 | 23.263 | 170.000 | 1,585,873.0 | 655,794.0 | 0.414 | |
| Niagara | Urban | 11 | 5,632.0 | 470.502 | 26.335 | 1.600 | 216,469.0 | 176,175.0 | 0.814 | |
| Oneida | Urban | 14 | 1,850.0 | 354.963 | 6.120 | 0.805 | 234,878.0 | 164,428.0 | 0.700 | |
| Onondaga | Urban | 11 | 16,168.0 | 446.781 | 29.269 | 3.000 | 467,026.0 | 415,876.0 | 0.890 | |
| Ontario | Urban | 11 | 2,395.0 | 366.661 | 6.056 | 0.500 | 107,931.0 | 66,255.0 | 0.614 | |
| Orange | Urban | 31 | 2,800.0 | 631.541 | 5.203 | 0.400 | 372,813.0 | 221,955.0 | 0.595 | |
| Orleans | Rural | 4 | 3,948.0 | 473.379 | 6.938 | 1.375 | 42,883.0 | 15,006.0 | 0.350 | |
| Oswego | Rural | 12 | 1,570.0 | 278.017 | 5.691 | 0.375 | 122,109.0 | 46,685.0 | 0.382 | |
| Otsego | Rural | 3 | 1,924.0 | 538.041 | 3.576 | 0.750 | 62,259.0 | 22,298.0 | 0.358 | |
| Putnam | Urban | 11 | 875.0 | 858.361 | 0.783 | 0.170 | 99,710.0 | 13,735.0 | 0.138 | |
| Queens | Urban | 4 | 621,853.0 | 6968.974 | 66.045 | 90.000 | 2,230,722.0 | 2,199,116.0 | 0.986 | |
| Rensselaer | Urban | 7 | 1,641.0 | 460.980 | 4.599 | 0.240 | 159,429.0 | 99,356.0 | 0.623 | |
| Richmond | Urban | 2 | 238,526.0 | 3802.959 | 74.714 | 49.950 | 468,730.0 | 468,730.0 | 1.000 | |
| Rockland | Urban | 6 | 24,602.0 | 977.051 | 26.747 | 4.900 | 311,687.0 | 311,687.0 | 1.000 | |
| Saratoga | Urban | 7 | 2,030.0 | 596.936 | 2.847 | 0.550 | 219,607.0 | 167,687.0 | 0.764 | |
| Schenectady | Urban | 5 | 14,825.0 | 752.507 | 13.852 | 1.500 | 154,727.0 | 115,856.0 | 0.749 | |
| Schoharie | Rural | 7 | 589.0 | 307.692 | 1.914 | 0.200 | 32,749.0 | 7,982.0 | 0.244 | |
| Schuyler | Rural | 1 | 3,656.0 | 402.025 | 9.094 | 1.200 | 18,343.0 | 3,656.0 | 0.199 | |
| Seneca | Rural | 5 | 3,080.0 | 263.137 | 9.580 | 0.700 | 35,251.0 | 17,803.0 | 0.505 | |
| St Lawrence | Rural | 23 | 422.0 | 262.214 | 2.877 | 0.100 | 111,944.0 | 72,824.0 | 0.651 | |
| Steuben | Rural | 11 | 1,893.0 | 484.909 | 3.970 | 0.500 | 98,990.0 | 38,361.0 | 0.388 | |
| Suffolk | Urban | 30 | 2,124.0 | 1159.271 | 2.459 | 0.400 | 1,493,350.0 | 397,069.0 | 0.266 | |
| Sullivan | Rural | 22 | 376.0 | 137.768 | 4.256 | 0.415 | 77,547.0 | 25,187.0 | 0.325 | |
| Tioga | Rural | 5 | 3,750.0 | 499.033 | 8.514 | 1.000 | 51,125.0 | 14,601.0 | 0.286 | |
| Tompkins | Urban | 7 | 2,276.0 | 323.187 | 6.644 | 0.500 | 101,564.0 | 74,183.0 | 0.730 | |
| Ulster | Urban | 20 | 661.0 | 418.736 | 1.983 | 0.325 | 182,493.0 | 65,840.0 | 0.361 | |
| Warren | Urban | 5 | 1,440.0 | 258.699 | 3.640 | 0.300 | 65,707.0 | 27,096.0 | 0.412 | |
| Washington | Rural | 6 | 2,094.0 | 551.753 | 2.952 | 0.380 | 63,216.0 | 22,197.0 | 0.351 | |
| Wayne | Rural | 13 | 2,518.0 | 383.061 | 6.715 | 0.600 | 93,772.0 | 40,119.0 | 0.428 | |
| Westchester | Urban | 12 | 28,848.0 | 1113.786 | 23.805 | 5.500 | 949,113.0 | 858,537.0 | 0.905 | |
| Wyoming | Rural | 4 | 3,502.0 | 299.792 | 11.896 | 0.735 | 42,155.0 | 11,641.0 | 0.276 | |
| Yates | Rural | 3 | 1,549.0 | 400.825 | 2.922 | 0.300 | 25,348.0 | 7,545.0 | 0.298 | |
