# Supplementary material for: Using geographic information systems to link population estimates to wastewater surveillance data in New York State, USA
Source: PLOS Glob Public Health. 2023 Jan 26;3(1):e0001062. doi: 10.1371/journal.pgph.0001062 (PMC10021809; doi:10.1371/journal.pgph.0001062)
Supplement: Attachment — Submitted filename: Response to reviewers.pdf [file pgph.0001062.s004.pdf]

PGPH-D-22-01363

Using geographic information systems to link population estimates to wastewater surveillance data in New York State, USA
PLOS Global Public Health

Comments from the editor

Comment 1:
Please provide separate figure files in .tif or .eps format only and remove any figures embedded in your manuscript file. Please also ensure that all files are under our size limit of 10MB.
For more information about figure files please see our guidelines: LINK
https://journals.plos.org/globalpublichealth/s/figures
https://journals.plos.org/globalpublichealth/s/figures#loc-file-requirements

Author Response
We have prepared separate files for each figure.

Comment 2:
We have noticed that you have uploaded Supporting Information files, but you have not included a list of legends. Please add a full list of legends for your Supporting Information files after the references list.

Author Response
We thank the editor for their comments, we have added the list of legends to the paper after the references.

Comment 3:
Please review your reference list to ensure that it is complete and correct. If you have cited papers that have been retracted, please include the rationale for doing so in the manuscript text, or remove these references and replace them with relevant current references. Any changes to the reference list should be mentioned in the rebuttal letter that accompanies your revised manuscript. If you need to cite a retracted article, indicate the article's retracted status in the References list and also include a citation and full reference for the retraction notice.

Author response
We have reviewed our reference list and believe we have corrected any mistakes that may have been present.

Comments from Reviewer #1

Comment 1:
Abstract. Some areas of the United States do have centralized databases for geographic information on sewer systems. Do any other states have a fully centralized database? Add a

qualifier to the statement “there are no centralized databases for geographic information on sewer systems”

Author response
We have added a qualifier to the abstract that New York does not have a database.

Comment 2:
Line 40, add at the end of this line “or with the addition of pump stations.”

Author response
We have made this correction.

Comment 3:
Line 51, again need to qualify that you are talking just about NY here.

Author response
We have made this correction.

Comment 4: Was there any overlap in service areas identified? This could be in high precipitation events for example where one sewershed diverts to another one.

Author response
There was no overlap in the service areas we identified. Any overlapping systems were removed and only the “parent” sewershed was drawn. We have included a statement about this on page 10 line 184 of the track changes document.

Comment 5: Were any of your sewersheds CSO/SSO?

Author response
Yes, some systems were CSO systems and some were SSO systems.

Comment 6:
Authors should calculate high and low ranges for the counties studied of how many residents are on sewer, which indicates the total portion of the community captured by WBE samples.

Author Response
We have made these calculations and they are not included in Supplemental Table 1. We include reference to the range of population served on page 12 line 232 of the track changes document.

Comment 7:
I think one of the most useful parts of this work is not the population covered, but knowing the spatial "gap" in households without services in areas where it "should" be available. For example, this might be an old house with a failing septic tank or straight pipe, which in addition to excluding this house from WBE samples also poses a risk to human health and the environment of surface water and your system could highlight these houses for public health attention. Can you choose one or two areas and conduct this additional analysis for comparison?

> *Author Response*
> We thank the reviewer for this comment and we have given thought to homes on private systems that might be missed in WBE studies. This is a common issue and somewhat insurmountable considering that WBE is a community-based assessment. Examining individual or even groups of homes that are not on sewer might pose a potential infringement on privacy and we have therefore not conducted a study of that detail. New York State does consider these private parcels when geo-locating cases and hospitalizations linked to diseases tested in wastewater for epidemiology studies. Since this is outside the scope of this project, we have decided not to conduct this analysis.

Comment 8:
In many older mid-western cities, straight pipes remain in pockets of urban areas. How do you account for this in NY, if present.

> *Author response*
> For drawing all sewer systems, we did not exclude single properties not connected to sewer systems that were completely inside the polygon boundary we drew. In addition, knowledge of straight pipes would require advanced and detailed understanding of each community, which we were not able to do considering the time we had to complete this project. We have added a statement to the limitations about this on page 20 lines 367-371 of the track changes document.

Comment 9:
A table in the supplement giving an overview of population size and a list of the counties you were/were not able to include would be helpful.

> *Author response*
> We agree with the reviewer and we now include supplemental table 1 that lists each county, the number of systems, and other descriptive statistics from the project.

Comment 10:
Conclusion. How could the findings of this work be implemented across 2 or 3 jurisdictions? Is there a list of minimum information needed for this merge (Add a table)? What can be done within rural environments with limited digitization of sewer pipes (physical maps are only covered in the limitations)?

Author response

This is an excellent idea from the reviewer and we have added a section to the paper titled: “Advice for adapting these methods for other jurisdictions” beginning on page 18 of the track changes document. Here, we suggest ways to use our methods in other locations in the U.S. and abroad taking into account the challenges we faced and what others might do to overcome them.

Comment 11:

Finally, when you submit the corrected version, please do check thoroughly, in order to avoid grammar, syntax or structure/presentation flaws.

Author response

We have reviewed the paper for grammar and spelling errors and edited where appropriate.

Reviewer #2

Comment 1:

This is a very useful paper detailing a project in NY state delineating the boundaries of sewersheds using a variety of extant data. This paper would be very helpful for practitioners and researchers and the resulting sewershed data has been valuable for public health actions and research. The manuscript is clear and the methods area well described; Fig 1 is very nice.

I have two primary comments. First, I do not agree that the correlations between log(capacity) and population metrics provide a high degree of evaluating accuracy. I have provided more detailed comments below. If the correlations are with capacity and not log(capacity), I apologize for my misunderstanding.

Author response

The calculations were with log(capacity) and log(population) in all instances. We understand the reviewers concerns about this. We log transform our variables because they are right skewed meaning that tests for linear correlation might be inaccurate without transformation. We did test capacity and population variables without log transformation finding they correlated equally. Below we provide some example figures to support our decision. We have not revised our manuscript, and we retain the use of log transformed variables. We do include justification on page 9 line 177 explaining the skewness and report the not log transformed correlations on page 11 line 223. Further, average flow was only available for 210 locations. We compared this with discharge capacity finding they were highly correlated with each other, which means that using discharge capacity seemed appropriate.

Flow v. capacity

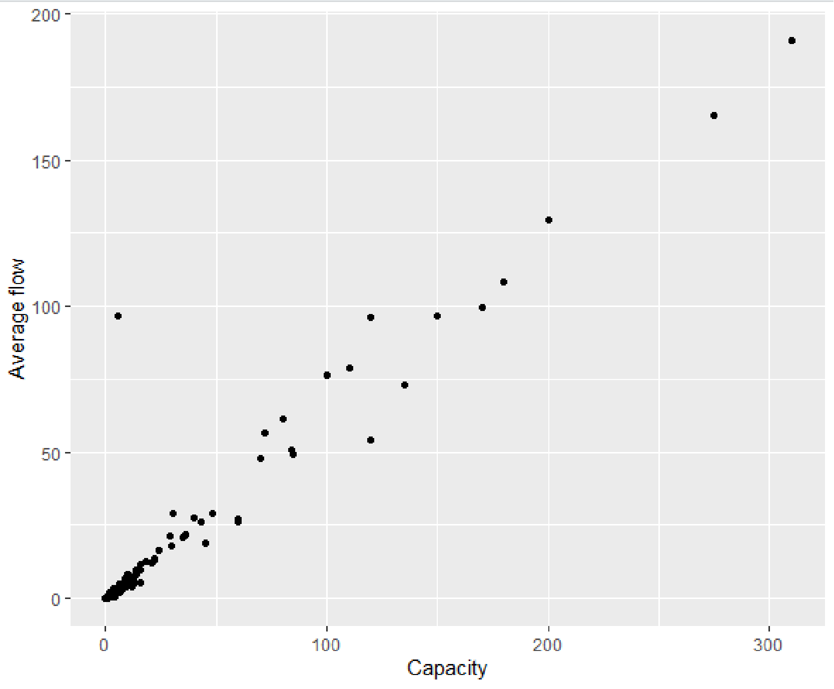

Capacity skewness

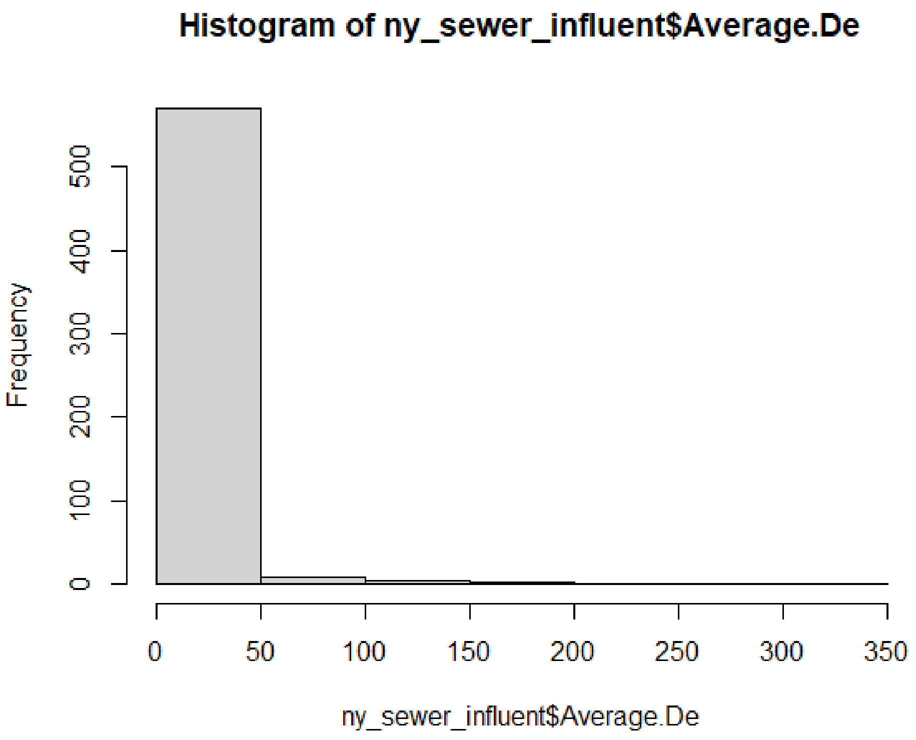

Correlations with untransformed variables

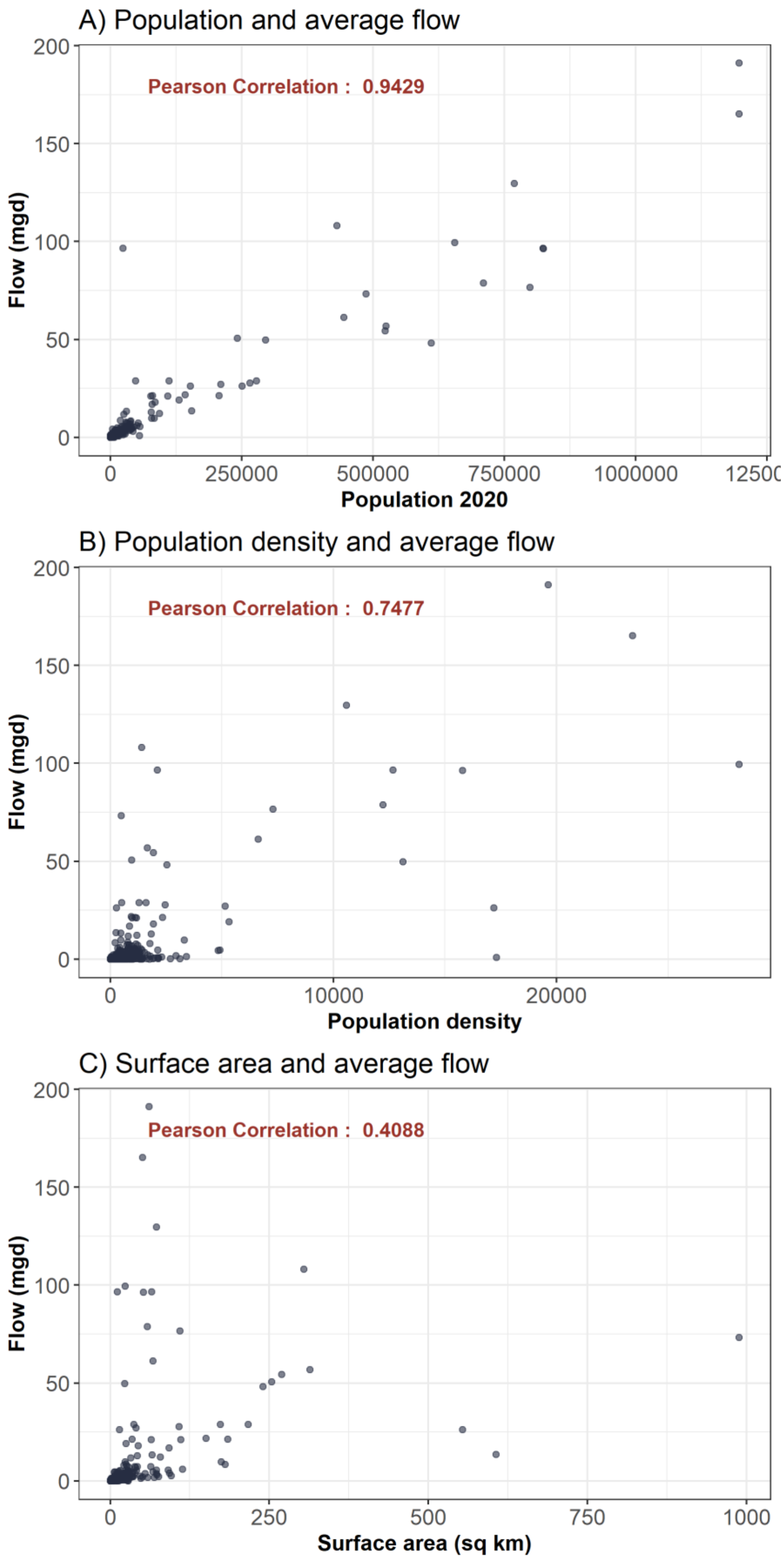
A) Population and average flow
Pearson Correlation : 0.9429
Flow (mgd)
Population 2020
200
150
100
50
0
0
250000
500000
750000
1000000
1250000
B) Population density and average flow
Pearson Correlation : 0.7477
Flow (mgd)
Population density
200
150
100
50
0
0
10000
20000
C) Surface area and average flow
Pearson Correlation : 0.4088
Flow (mgd)
Surface area (sq km)
200
150
100
50
0
0
250
500
750
1000

Comment 2:
I imagine that the authors have a great deal of knowledge about how accurate the resulting sewersheds are and the factors that result in having accurate or inaccurate boundaries. This leads to my second comment: this experience and a few other topics would be of great use to readers and should be covered in the discussion.

Author response
This is an excellent suggestion and along with feedback from the other reviewer, we have added a section titled: "Advice for adapting these methods for other jurisdictions" to the discussion starting on page 18.

Comment 3:
Line 37 – Consider replacing 'area' to 'extent'

Author response
We have made this change.

Comment 4:
Line 42 – Note that lift stations are also used to not only feed force mains, but to feed gravity flow sewers from a higher starting elevation.

Author response
This is an excellent point and we have added a statement about lift stations on page 2 line 44 of the track changes manuscript.

Comment 5:
Line 84 – where did the special district tables come from?

Author response
These data came from the parcel data as a companion dataset. We have added reference to this on page 4 line 89 of the track changes manuscript.

Comment 6:
Line 143: Did you use the same asymetric apportionment for the block groups as you used for the 2010 block populations? Please clarify.

Author response
We used the same methods for both block groups and blocks. We have clarified this on page 9 line 153 of the track changes manuscript.

Comment 7:
Line 154: It doesn't seem that this process was validation as you didn't appear to have a 'true' or gold standard boundary to compare to other derived boundaries, and when you did have a 'good' boundary, you used that. In some cases it sounds like you used a combination of methods. In addition it appears that you compared methods to get a sense of the reliability of your estimates. Comparing population estimates, which have inherent and non-random biases due to the apportionment methods, to the permitted flow may be a form of validation. However permitted flow is not actual average flow (which we have been able to get from most WWTPs). Were there any criteria used to determine if a sewershed appeared to be accurate based on a relationship between population and permitted flow?

> *Author response*
> We appreciate these questions from the reviewer. Average flow for the plants that we had data for correlated highly with discharge capacity and we felt using a variable that was complete for all data points was better than using the incomplete variable.
> We agree that the methods are less validation and more of an evaluation of results using what we know to identify any anomalies in our estimates. The criteria used for accuracy was difficult to determine and we relied on direction consultation with treatment plant operators where possible. This gave us confidence in how these boundaries should look for areas that we were unable to connect with a plant manager. We explain this starting on page 18 line 316.

Comment 8:
This is a relatively small point. I recommend that you re-word the section and characterize it as just QA. Reliability is a concept you seem to be describing in this section. Quality Control is more accurate as you are assessing the resulting product (the sewershed boundaries) rather than the efforts and protocols set up in the process of creating the sewersheds to assure high quality boundaries.

> *Author response*
> We have reworded this section to be about quality assurance.

Comment 9:
Line 170. I was initially confused by the 49.1% value. I would move the statistic down and incorporate somewhere after you have presented the context of having 638 sewersheds in total.

> *Author response*
> This is an excellent suggestion and we now lead the results with this explanation starting on page 10 line 181 of the track changes manuscript.

Comment 10:

Line 176. I suggest that you first discuss that of the initial 638 presumed WWTP/sewershed, some were duplicates or decommissioned leaving 638-46=592 actual WWTP. This better highlights that you were able to create sewersheds for all active WWTPS/sewersheds.

Author response

This is an excellent suggestion and we now lead the results with this explanation starting on page 10 line 181 of the track changes manuscript.

Comment 11:

Line 188. There are vast differences in population across NY so this isn't surprising. Variation in the number of sewershed per county by population or population density would be interesting and illustrate why and where there are larger vs smaller systems. There needs to be a table summarizing the size and population distribution of sewersheds, perhaps stratified by urban/rural status of the county.

Author response

This was a good suggestion from the reviewer. We now include a summary of urban and rural counties in Table 3 on pages 14-15 and a supplemental table that breaks this out by county. Interestingly, there was not much correlation between the number of treatment plants and whether the county was urban or rural. Indeed urban counties ranged in number of systems between 1 and 30 suggesting that there are still old and decentralized systems across the state even in high population density counties.

Comment 12:

Line 194. Consider using 'sewershed area' rather than surface area.

Author response

We have made this edit throughout the paper.

Comment 13:

Line 192 – 198. If the reported p-values are those reported by most software packages, the null hypothesis is that the correlation=0, s a p-value<0.05 indicates that the correlation is significantly different from 0, not a very meaningful statistical test. As these are descriptive statistics I recommend removing references to these p-values.

Author response

We agree with the reviewer and we have dropped all reference to p values in the paper.

Comment 14:

Figure 3 indicates that the correlations were between log(capacity) and sewershed characteristics (log base 10 I am assuming). However this paragraph and Table 2 imply that the values were not log transformed. Please clarify in the text and table. Please provide a rationale for the use of log(capacity). Non-household sewer inputs notwithstanding, there should be a direct correlation

between the flow and population. As capacity is being used as a surrogate for flow, I think actual capacity should be used. If thee a rationale that I have missed, lease clarify.

> *Author response*
> Please see our response above to comment 1. Additionally, we have edited our paper to now reference log capacity and log population.

Comment 15:
Figure 2. Please label the color bar. Is yellow high density or low? I recommend reversing this so higher density population is represented by the darker color.

> *Author response*
> We have edited this figure to indicated high and low as well as swapping the colors.

Comment 16:
Figure 3. Revise the title to indicate that these are scatter plots, not correlations.

> *Author response*
> We have made this correction.

Comment 17:
Please address industrial and other non-household inputs and how they might be incorporated into this type of data system. In addition travel distance/travel time may be very helpful for understanding observed concentrations at the WWTP for non-conservative substances or infectious organisms that may decay in the system. This may be useful to note.

> *Author response*
> We have added a statement to the limitations about industrial discharges and sewer travel times on pages 20-21 lines 389-393 in the track changes manuscript.

Comment 18:
Line 213. The correlations did not involve the polygons, just permitted discharge capacity and population measures. Note my comments above regarding the use of log(capacity). Note that not all of the correlation coefficient would be considered high (0.57). It would be better to say that the majority of correlations were high, rather than saying ‘all’ and then saying in the next sentence that there was an exception.

> *Author response*
> We have edited this section to be more careful about what we call “high” correlations. Please see pages 11 and 12 of the track changes manuscript.

Comment 19:
I don’t agree that a high correlation between log of permitted discharge capacity provides evidence that the boundaries were accurate. It does perhaps show that the different methods may

be comparable, however, there was likely relationships between the size of the system and the type of data used to create the sewersheds. This would be a useful result to present. At a minimum the text should consistently reflect that the correlations and relationships were related to the log of permitted capacity.

Author response
We have edited the paper to reference log capacity. In addition, we have added a column to Table 2 on page 14 of the track changes document that includes the median capacity for each method. Interestingly, the provided data was actually among the smaller plants with the larger plants actually providing maps instead. The smallest systems were from DANC records, which is not surprising considering that the North Country Region in New York has lower population density than the other regions.

Comment 20:
Line 273: It would be useful to see the distribution of populations in a table, as noted above. Did you get the same population estimates using the block group data from the ACS? These populations are unrealistically small and this represents about 10% of your sewersheds.

Author response
We have added Table 3 that summarizes rural and urban population information as well as supplemental table 1 that includes details on each county's population on sewer. While we agree that the estimates for the small sewersheds below 100 might be less accurate, we do not agree that these are unrealistic. We have several sewer systems that serve small neighborhoods and in some cases single streets with a dozen or fewer homes. We have added this clarification to the paper on page 20 line 378 of the track changes manuscript.

Comment 21:
Line 279. Consider "These data will require updates in the future given the potential for …"… There are a few items that readers would find useful. What was the effort need to complete this project? About how many hours were needed for large and small systems or by type of data provided? What is your assessment of accuracy and in what situations do you think that the boundaries are not reliable? How might the process be improved?

Author response
These are excellent suggestions from the reviewers and we have added a section describing the process and time used to create these data to page 19 starting on line 345 of the track changes manuscript.

Comment 22:
Further, the limitations of having only permitted discharge capacity (which may be different than actual discharge capacity/design capacity) needs to be addressed. It is greater than actual flow and this may vary with age of system and other factors. This affects the correlation coefficients. This is an important limitation to be discussed.

Author response
We have added a statement about this to the limitations on page 20 line 364 of the track changes document.
